# Structure, Function, and Modulation of γ-Aminobutyric Acid Transporter 1 (GAT1) in Neurological Disorders: A Pharmacoinformatic Prospective

**DOI:** 10.3389/fchem.2018.00397

**Published:** 2018-09-11

**Authors:** Sadia Zafar, Ishrat Jabeen

**Affiliations:** Research Center for Modeling and Simulation, National University of Sciences and Technology, Islamabad, Pakistan

**Keywords:** γ-aminobutyric acid (GABA), GABA transporters (GATs), homology modeling, molecular dynamics (MD), QSAR

## Abstract

γ-Aminobutyric acid (GABA) Transporters (GATs) belong to sodium and chloride dependent-transporter family and are widely expressed throughout the brain. Notably, GAT1 is accountable for sustaining 75% of the synaptic GABA concentration and entails its transport to the GABA_A_ receptors to initiate the receptor-mediated inhibition of post-synaptic neurons. Imbalance in ion homeostasis has been associated with several neurological disorders related to the GABAergic system. However, inhibition of the GABA uptake by these transporters has been accepted as an effective approach to enhance GABAergic inhibitory neurotransmission in the treatment of seizures in epileptic and other neurological disorders. Here, we reviewed computational methodologies including molecular modeling, docking, and molecular dynamic simulations studies to underscore the structure and function of GAT1 in the GABAergic system. Additionally, various SAR and QSAR methodologies have been reviewed to probe the 3D structural features of inhibitors required to modulate GATs activity. Overall, present review provides an overview of crucial role of GAT1 in GABAergic system and its modulation to evade neurological disorders.

## Introduction

Transporters or solute carriers are membrane bound proteins involved in the transport of signaling molecules such as ions, nutrients, and various amino acids. The transport of the impermeant solutes against concentration gradient is ATP mediated. Among these transporters, solute carrier (SLC) transporter is one of the major class of human transport proteins that act as symporters, antiporters, exchangers, and are classified into 55 families on the basis of variation in structural elements and biological functions (Hediger et al., 2013). However, SLC6 transporters (the sodium- and chloride-dependent neurotransmitter transporter family) including γ-Aminobutyric acid transporters (GATs), norepinephrine transporter (NET), dopamine transporter (DAT), and serotonin transporter (SERT) encoded by SLC6A1-4 genes in humans are specifically known to be important for efficient neuronal synaptic transmission hence providing neurotransmitter homeostasis in the central nervous system (CNS) (Ben-Yona et al., 2011; Kristensen et al., 2011; Pramod et al., 2013).

NET, DAT, and SERT are further classified under monoamine transporters whereas GATs are amino acid transporters also known as GABA neurotransmitter transporters (Singh et al., 2007). The mammalian GATs are categorized into four subtypes, GAT1-3 and BGT1 (Betaine GABA transporter) with respect to their amino acid sequence and pharmacological properties (Conti et al., 2004; Besedovsky et al., 2007; Parpura and Haydon, 2008). Briefly, GAT1 and GAT3 subtypes accounts for major proportion in CNS. Peculiarly, GAT1 is mainly expressed throughout the brain in neurons (Jin et al., 2011); specifically at the presynaptic terminals of the axons and also in minute concentration in ganglia (Besedovsky et al., 2007; Rego et al., 2007; Wilson, 2011) whereas GAT3 is mainly localized at the perisynaptic astrocytes (Melone et al., 2015). Nevertheless, GAT2/BGT1 are expressed in the liver, kidney, meninges as well as at the blood brain barrier (BBB) (Takanaga et al., 2001; Zhou and Danbolt, 2013).

The imbalance in homeostasis of various ions including Na^+^ and Cl^−^ due to dysregulation of monoamine transporters at neuronal cells is widely associated with the modulation of anxiety, appetite, mood, attention, depression, and aggression etc (Singh et al., 2007). However, dysregulation of GATs (amino acid transporters), under pathological conditions results in extra removal of GABA neurotransmitter from the synapse thereby leads to severe mental illnesses like Parkinson's disease, Alzheimer, schizophrenia, and seizures in epilepsy (Hack et al., 2011; Schaffert et al., 2011). Generally, imbalance in GABAergic neuronal circuits due to lowered expression of glutamic acid decarboxylase (GAD), a key enzyme for the conversion of excitatory neurotransmitter glutamate into inhibitory neurotransmitter GABA in the presynaptic neuron, is affiliated with onset of epileptic seizures (Gupta, 2011). Moreover, decreased levels of GABA transaminase (GABA-T), a known catabolizer of GABA into succinic semialdehyde, are also profound in choreoathetosis, encephalopathy, hypersomnolence, Alzheimer's disease, and epilepsy. The lower GABA-T propagates the higher levels of GABA in the intraneuronal cytoplasm that causes certain pathological/psychiatric and pharmacological effects (Sadowski, 2003). Markedly, as GATs are in direct contact with the GABA neurotransmitter in the extracellular space therefore, of all the stated GABAergic system components, GATs have attained significant importance for maintaining concentration gradient during abnormal conditions (Yamashita et al., 2005).

Notably, GAT1 is mainly involved in the GABA binding and transport from the cytoplasm to extracellular space (reverse mode) and from the extracellular space back into the cytoplasm (forward mode). Thus, malfunctioning of GAT1 may provoke delay in communication with the post-synaptic GABA receptors (Scimemi, 2014) which may result in various neurological disorders (Hack et al., 2011; Schaffert et al., 2011). Given the pivotal role of GAT1 in GABAergic transport mechanism, it has been recognized as potential therapeutic target for decades (Bialer et al., 2007). Therefore, inhibition of GABA re-uptake transport [either through clinically tested GABA reuptake inhibitors (GRIs) or GAT1 selective antiepileptic FDA approved drug Tiagabine (Trimble and Schmitz, 2011)] to block the extra removal of GABA from synapse is the most accepted strategy to maintain a concentration gradient and normal activity of GABA at the synaptic clefts (Zhou et al., 2007, 2009; Krishnamurthy and Gouaux, 2012; Scimemi, 2014). Thus, this review highlights the structural and functional properties of GAT1 and also elucidates the important 3D structural features of its antagonists. Additionally, pharmacoinformatics strategies including quantitative structure-activity relationship (QSAR), pharmacophore modeling, homology modeling, molecular docking, and molecular dynamics (MD) studies have been highlighted to underscore the overall binding hypothesis of human γ-Aminobutyric acid transporter (hGAT1) modulators.

### Mechanism of action of GATs

**Figure 1:**
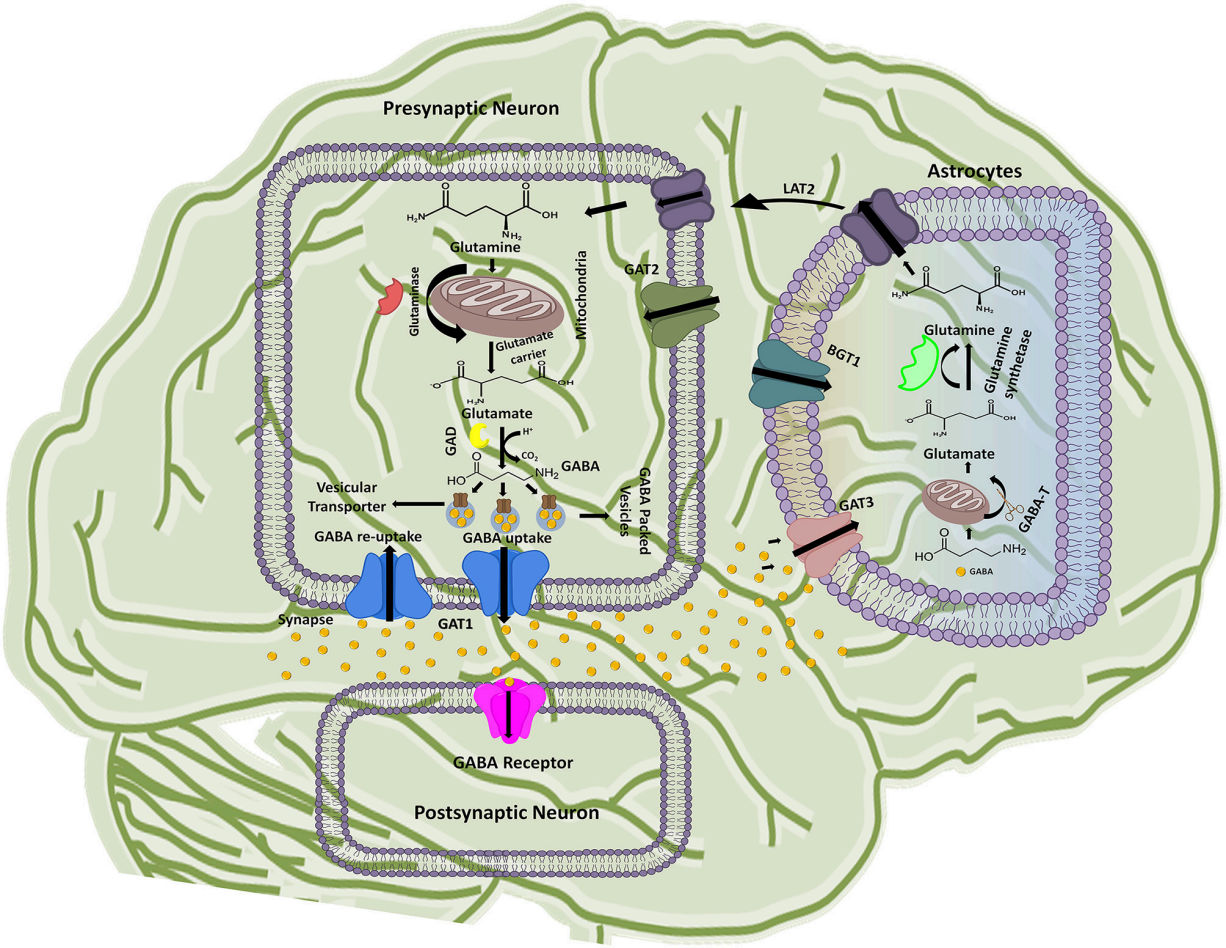
Schematic illustration of GABA synthesis, release, uptake, re-uptake, and metabolism in the CNS. In the presynaptic neuron, conversion of glutamine to glutamate followed by the GABA synthesis is represented along with the respective catalytic enzymes and transporters. GABA receptor present on the post-synaptic neuron receives the signals from the presynaptic neuron while astrocytes functions to metabolize the extra amount of GABA in the synaptic cleft and process it to glutamine. The processed glutamine is transported to the presynaptic neuron with the help of Linker for activation of T-cells family member 2 (LAT2) transporter. Thus, synthesis of GABA in presynaptic neuron is started again on the arrival of new signal.

The GABAergic mechanism starts with the conversion of an excitatory neurotransmitter Glutamate into the inhibitory neurotransmitter GABA by an enzyme glutamate decarboxylase (GAD) in the mature mammalian brain (Gropper and Smith, 2012). This conversion is followed by GABA packing and release into the synaptic vesicles. The vesicle's uptake priority is given to the newly synthesized GABA in lieu of the preformed GABA. However, the underlying mechanism of such priority supply of glutamate to the inhibitory synaptic terminals and to maintain the vesicles content with fresh GABA formation is not completely understood till date (Stafford et al., 2010). Hence, it has been advocated that GABAergic neuronal networks are mainly responsible for the synthesis and release of vesicular packed GABA along with the respective ions from presynaptic nerve terminals into the synaptic cleft down their electrochemical gradient as shown in Figure 1 (Deidda et al., 2014). However, regulation of GATs functioning is dependent on a wide variety of signaling cascades including “second messengers” (such as pH, kinases, arachidonic acid, and phosphatases) and “synaptic proteins” (such as syntaxin) that play crucial role in functional modulation of GATs (Law et al., 2000). For instance, phosphorylation of tyrosine residues of GAT1 by tyrosine kinase helps in mediating its GABA transport function (Law et al., 2000; Wang and Quick, 2005). Various reports indicates that upon activation released vesicular GABA from the presynaptic neurons is taken up by GAT1 and transported to the GABA receptors that are present on the post-synaptic terminals of the dendrites across the synapse, as synaptic GABA does not undergoes enzymatic breakdown (Figure 1) (Gonzalez-Burgos, 2010).

GABA is known as a key player of regulating plasticity and inhibiting anxiety in eukaryotes (Brady et al., 2018). However, the action of GABA is terminated to maintain its concentration in the synapse. In this prospect GAT1 initiates about 80% GABA re-uptake into presynaptic neurons (forward mode) from where it releases again (reverse mode) when require. However, around 20% of the GABA molecules are metabolized into glutamine after transportation to the glial astrocytes by GAT3 and thus, are not available for the neuronal release (Figure 1). Hence, the next cycle begins with the conversion of glutamine to glutamate followed by conversion into GABA once again (Parpura and Haydon, 2008).

**Figure 2:**
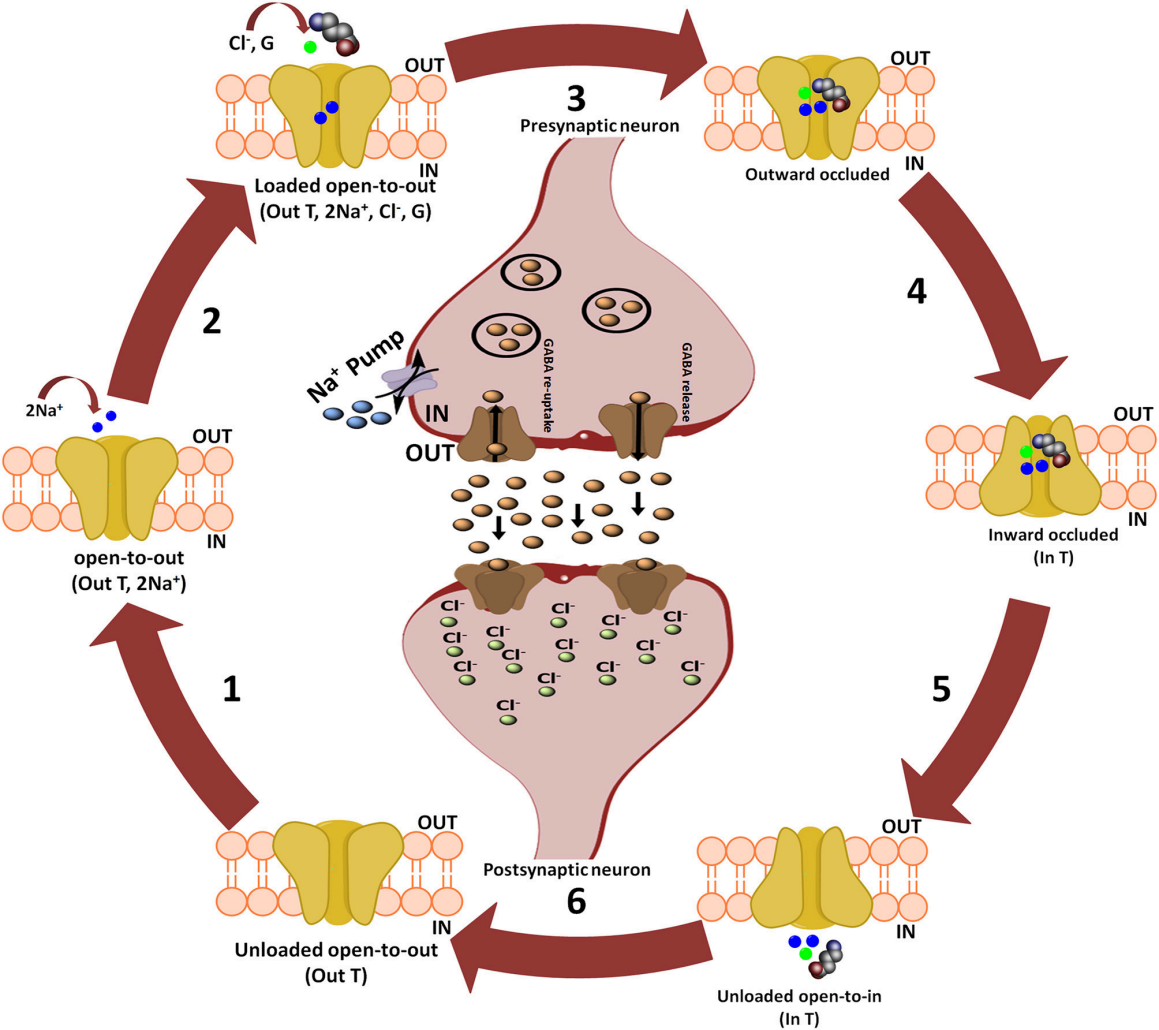
Translocation cycle of GAT1. Steps 1–4 of translocation cycle represent the open-to-out conformation of hGAT1 which involves the co-transport of two Na^+^ ions along with a Cl^−^ ion and GABA (also known as forward mode of reaction). In forward mode, net influx of two Na^+^ ions through GAT1 results in the increased concentration of Na^+^ ions in the cytoplasm. To maintain the intracellular Na^+^ ions concentration, Na^+^ pump present on the presynaptic neuronal membrane effluxes the Na^+^ ions into synaptic cleft to maintain the concentration gradient. Step 5 represents open-to-in conformation of hGAT1 (also known as reverse mode of reaction). In reverse mode functioning of Na^+^ pump is opposite to that of the forward mode. In step 6, the hGAT1 becomes empty again to begin a new cycle.

Further, Rosenberg and colleagues conferred the translocation cycle of GAT1 as explained in Figure 2. Briefly, GAT1 adopts three distinct conformations i.e., open-to-out, occluded-out, and open-to-in conformations. When empty, GAT1 faces the extracellular medium (out T) to which two Na^+^ ions bind (step 1). Na^+^ ions stabilize the binding of substrate in the protein core. In a follow-up step, GABA (G) and a Cl^−^ ion bind with the transporter so that the transporter becomes loaded. However, theoretical and computational studies have revealed that prokaryotes do not require chloride (Cl^−^) ion for the transport (Scimemi, 2014). Since, it is necessary in eukaryotic mammals for the compensation of positive charge induced by the co-transport of Na^+^ ions during GABA translocation step to maintain the membrane potential (Rosenberg and Kanner, 2008). In step 3, fully loaded transporter adopts a seal conformation which does not allow the release of ions and/or substrate to either intracellular (cytoplasm) or extracellular (synapse) medium until or unless it changes its conformation. Subsequently loaded transporter becomes inward facing (step 4) and then GABA and co-transported ions are released into the cytoplasm (step 5). The empty inward facing transporter (in T) transits to again occlude its binding pocket and thus resume outward facing empty transporter configuration (step 6). Hence, a new translocation cycle begins again (Rosenberg and Kanner, 2008).

## Structural and functional homologs of GAT1

Various attempts have been made to determine the crystal structure of GATs in humans. However, struggles remained unsuccessful due to the unavailability of appropriate quantities of pure and stable transporter proteins. In prokaryotes, availability of X-ray crystal structure of *Aquifex aeolicus* leucine transporter (*Aa*LeuT, PDB ID: 3F3A) that shares remarkable functional similarity and about 25% sequence similarity with eukaryotic GATs (Kristensen et al., 2011) has augmented the research efforts to elucidate the structure and function of human GATs (hGATs). Moreover, crystal structure of dopamine transporter (DAT, PDB ID: 4XP4) in open-to-out conformation in *Drosophila melanogaster* (Wang et al., 2015) (that shares 46% sequence identity with GAT1) serves as a good template for the molecular modeling of the tertiary structure of GATs.

Though, detailed insights into functional inhibition mechanism of GATs remained exclusive till date. Yet, inhibition of hGAT1 translocation cycle at any of the three distinct conformational states of hGAT1 (open-to-out, occluded-out, or open-to-in) to inhibit the extra removal of GABA neurotransmitter has been reported by various authors in the past. However, open-to-out and occluded-out conformations are mostly targeted (Beuming et al., 2006; Skovstrup et al., 2010). It has been demonstrated that inhibition of open-to-out conformation obstructs hGAT1 to acquire occluded-out conformation responsible for translocation of substrate GABA and co-transported ions (Baglo et al., 2013). As the transport of the substrate is mediated through two binding sites i.e., S1 and S2, none of the *Aa*LeuT or dDAT crystal structures were solved with a bound ligand at S2 site until 2008. Briefly, S2 site is known as a low affinity and temporary occupied region for the ligands, as they finally move toward the S1 site from the extracellular vestibule (Quick et al., 2009).

Later, researchers were successful in elucidating the importance of S2 site through impairment of symporter activity (i.e., release substrate molecule to the S1 site) in a mutagenesis study conducted on hGAT homolog, *Aa*LeuT. The substitution of *n-*octyl-D-glucopyranoside (OG) detergent along with the substrate at S2 site trapped the transporter in its open-to-out conformation due to its inhibitor like effect on activity thereby blocked the translocation of substrate to S1 site; subsequently led to the inhibition of occluded transport conformation (Quick et al., 2009). Moreover, in case of dDAT the binding of inhibitor cocaine at the S2 site in open-to-out conformation during pathogenic conditions induced the conformational change in the binding site of dDAT to facilitate its translocation to S1 site. The binding of the cocaine to S1 site resulted in the blockade of conformational shift from open-to-out to occluded-out conformation (Clementi and Fumagalli, 2015; Wang et al., 2015) which ultimately results in the inhibition of dopamine transport into the cytoplasm; eventually leading to the neuromodulation to overcome anxiety and depression.

### Topology and physiological properties of GAT1

**Figure 3:**
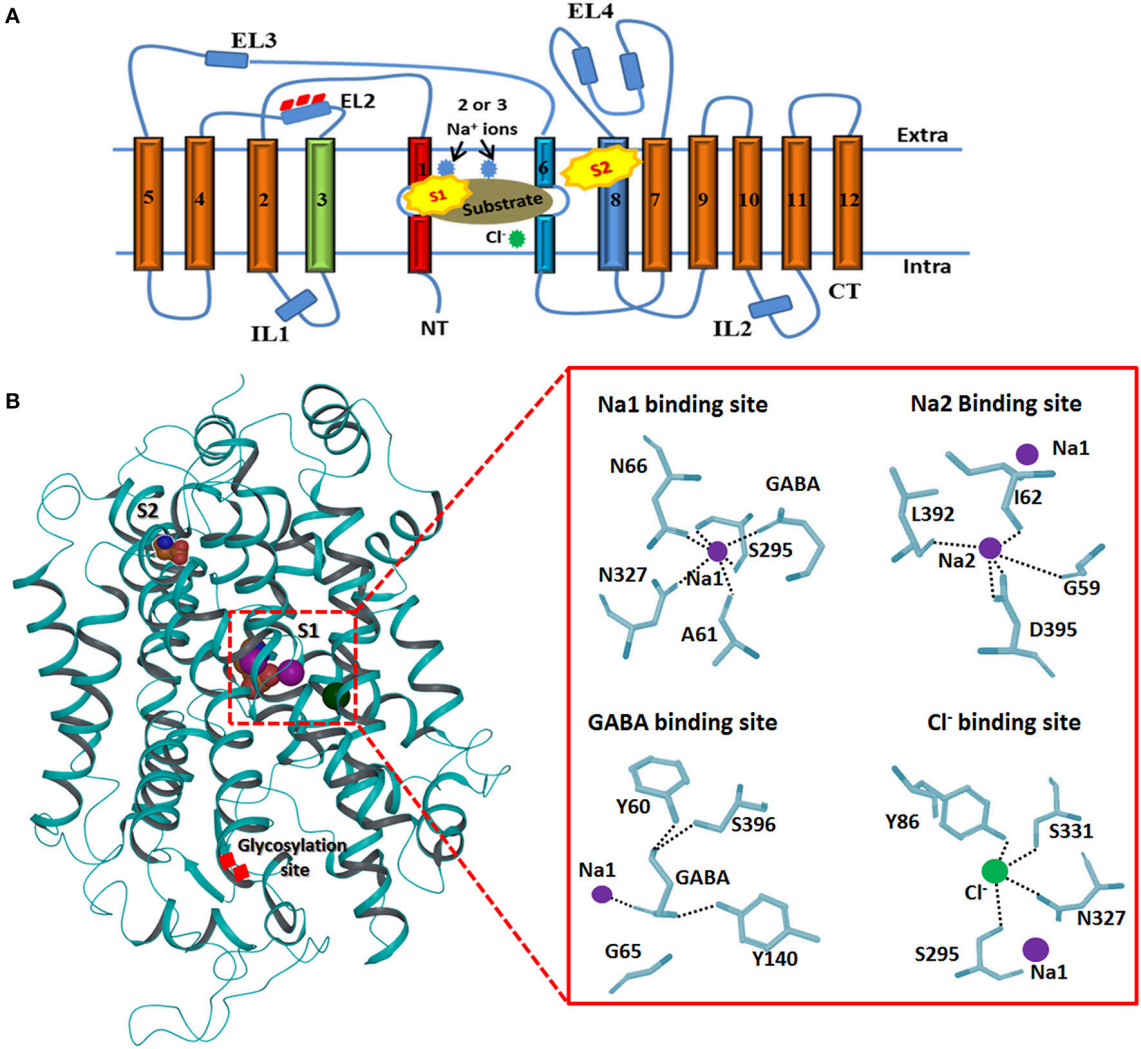
Generalized membrane topology of GATs. **(A)** The TM segments 1 and 6 (shown in red and light blue) along with the TM segments 3 and 8 (shown in green and dark blue) are involve in the formation of cylindrical ring for binding of the substrate and co-transported ions. Rests of the TM segments are shown in brown. Interconnecting intra- and extracellular loops (ILs and ELs) are represented with dark blue color. S1 and S2 represent the primary and preliminary binding sites of the substrate. **(B)** 3D structure of hGAT1 possessing substrate (S1 and S2), ions binding sites and glycosylation sites. Purple sphere shows sodium ions and Cl^−^ ion is represented with green color. An enlarge view of GAT1 residues interaction profile of GABA and all three ions is presented on right side.

The topology of GAT1 was first determined with the help of hydropathy plots that assists in structure elucidation of rest of the members of GATs as they share significant similarity (>50%) (Cummings et al., 2009). Hydropathy plots allow the identification of the domains which are soluble or insoluble i.e., charged or uncharged amino acids regions, respectively, over the length of protein sequence. Thus, sequence and structural inspection of electron microscopic, epitopic and X-ray crystallographic studies delineates that GATs consists of 12 transmembrane (TM) segments with N- and C-terminus facing cytoplasm as shown in Figure 3. Overall, GATs encompasses two pseudo repeats of helices i.e., TM1-TM5 and TM6-TM10. Moreover, TM segments 1, 3, 6, and 8 are majorly involved in the upholding of ions and substrate in GATs (Figure 3). Mutagenesis studies have provided detailed insights into some structural aspects of the defined topology that includes identification of N-glycosylation sites that fall in the hydrophilic extracellular loop (EL2) in between TM3 and TM4 segments (Masuda et al., 2008) whereas phosphorylation occurs in the intracellular loops (IL) of GATs with the help of tyrosine kinases (Bennett and Kanner, 1997). Moreover, mutagenesis studies have showed that removal of these glycosylation sites may result in the reduced GABA uptake activity however, malfunctioning of tyrosine kinases involves the redistribution of GATs from the cell surface to intracellular locations (Masuda et al., 2008; Jin et al., 2011). Arbitrary, GATs require transportation of an extracellular Cl^−^ ion along with Na^+^ ions and a GABA molecule (substrate) per transportation step as shown in Figure 3 (Reichenbach and Bringmann, 2010). However, stoichiometry of Na^+^:Cl^−^:GABA transport for GAT1, GAT2, GAT3, and BGT1 is 2:1:1, 2:1:1, ≥2:2:1, and 3:1(or 2):1, respectively (Loo et al., 2000; Dalby, 2003). In general, GABA molecule is zwitterion therefore, GATs propagates a net influx of one positive charge per transport step (Lu and Hilgemann, 1999).

#### Substrate and Na^+^ ions binding sites in hGAT1

Briefly, TM1 and TM6 segments contain unwound regions hence separating them as TM1a, 1b, 6a, and 6b. Moreover, I62 and G63 residues in the unwound regions adopt an extended conformation to link the TM1a-b segments whereas G307 to G311 are involve in linking TM6a-b segments. TM1 and TM6 segments, harboring the highest percentage of conserved residues, run in opposite direction. These two TM segments in their unwound regions along with TM3 and TM8 form the inner cylindrical ring (S1 binding site) which upholds the two Na^+^ ions and substrate binding site (Figure 3B). Amino acid residues G59, A61, I62, L64, G65, Y60, N66 (of TM1), Y140 (of TM3), S305, G307 (of TM6), N327 (of TM7), L392, D395, S396, L402, and S406 (of TM8) are known to be involved in pocketing Na^+^ ions and substrate in water depleted binding site of hGAT1 (Yamashita et al., 2005). However, S2 site is the preliminary allosteric site in the extracellular vestibule at which either substrate or inhibitor molecule binds. It mediates the release of Na^+^ ions and substrate to primary site (S1), thus, enables the sodium coupled GABA (substrate) symporter activity (Quick et al., 2009).

#### Cl^−^ ion binding site

The X-ray crystal structure of *Aa*LeuT does not encompass Cl^−^ ion. However, the uneven estimate of its binding in *Aa*LeuT is in the EL2 which is ~20Å away from the binding pocket (S1 site). Thus, the transport is considered as Cl^−^ independent transport (Forrest et al., 2007). In comparison, eukaryotic neurotransmitter transporters are Cl^−^ dependent and R69 is known to be a crucial residue in Cl^−^ ion binding during the transport. Moreover, replacement of any other residue with R69 especially charged residues abolishes the Cl^−^ ion binding hence obstructs the substrate transport (Lajtha and Reith, 2007).

Additionally, the structural analysis of SERT, one of the members of neurotransmitter transporters that share significant similarity with GATs, emphasized that Y121, S336, N368, and S372 interact through carbonyl oxygen and amide nitrogen with Cl^−^ ion in eukaryotes. The corresponding residues in prokaryotes are Y47, T254, N286, and E290 (Krogsgaard-Larsen et al., 2016). However, mutagenesis studies of S372 (corresponding E290 residue in prokaryotic *Aa*LeuT) with alanine, cysteine, glutamate and aspartate, and N368 (corresponding residue N286 in *Aa*LeuT) with aspartate inhibit the Cl^−^ ion mediated transport (Forrest et al., 2007). Later on, Kristian identified that Cl^−^ ion is important for the translocation of substrate (GABA in eukaryotes) against the concentration gradient by compensating the positive charges (Na^+^ ions). Thus, the specific residues of hGAT1 known for Cl^−^ ion dependence and selectivity are Y86, Q291, S295, N327, and S331 (Figure 3B) (Krogsgaard-Larsen et al., 2016).

Along with the substrate transport, the ions movements through neurotransmitter transporters also play a significant role in inducing conformational change in the TM helical segments of the binding pocket. Generally, in open-to-out conformation of transporter, encompassing the bound Na^+^ ions in the active site (S1), extracellular gates are relatively thin and remain open. However, substrate binding induces slight conformational changes in the extracellular regions of the TM1, TM2, TM6, and EL4 (Krishnamurthy and Gouaux, 2012). The functional role of EL4 is well-established in sealing of the binding site thereby leading to the occluded-out conformation (Gether et al., 2006). Upon release of Na^+^ ions into the cytoplasm (open-to-in state), the re-shifting of TM segments 1, 2, 5, 6, and 7 induces a major conformational change in the transporter structure once again. Furthermore, intense changes in the hinge region of TM1a and extracellular vestibule of EL4 i.e., bending and occlusion, respectively occurs. This allows the formation of thick extracellular and thin intracellular gates therefore, blocking the access of water in the binding cavity and permit access to binding site from the cytoplasmic face (Krishnamurthy and Gouaux, 2012).

Bio-physiologically, GAT1 encompass four basic properties thoroughly determined by [^3^H] GABA uptake assays performed on rats: (i) GAT1 have strong affinity for GABA molecules as a substrate at low micromolar concentration (Guastella et al., 1990), (ii) the increase rate of GABA uptake in the presence of K^+^- selective ionophore valinomycin help in the determination of the fact that this transport is voltage dependent across the membrane (Kanner, 1978; D'adamo et al., 2013), (iii) replacement of Na^+^ ion with other cations e.g., Li^+^, K^+^, Tris^+^ may affect the transport mechanism thus, suggesting Na^+^ ion crucial for the transport (Iversen and Neal, 1968; Nascimento et al., 2013), and (iv) the GABA transport requires electrochemical gradient of Na^+^ ion which is generated by Na^+^/K^+^ ATPase activity (Guastella et al., 1990; Hertz et al., 2013).

Although, GABA is now established as a major inhibitory neurotransmitter in the vertebrate brain (Tritsch et al., 2016), GABA presence in the CNS was not fully determined until 1975. However, during last 40 years a tremendous progress has been made to identify its role in CNS. In this regard, a number of experiments have been conducted on mice and crustacean models specifically in crayfish that helped in defining the role of GABA in GABAergic neuronal system mediated inhibition processes (Bowery and Smart, 2006). From more than 65 years, mutagenesis studies and wet lab experiments have been carried out to understand the functional relevancy of amino acid residues, quantitative measure or qualitative assessment of functional activity, presence or amount of the target (site/protein/chemical). Hereof, several biochemical, pharmacological, and physiological studies have shown determinable effects in comprehending GABAergic interneurons system and its use in treatment of epilepsy. For example, numerous studies have been conducted on activity of GAD enzymes, binding of GABA to post-synaptic GABA_A_ receptors, percentage reduction in GABA mediated inhibition, presence of GABA in brain tissue and cerebrospinal fluid (CSF) (Treiman, 2001). Hitherto, a huge number of tested acquired and genetic animal models have shown a clear evidence of abnormalities in GABA regulation in interneurons system (Horton et al., 1982; Olsen et al., 1985; Peterson et al., 1985; Roberts et al., 1985; King and Lamotte, 1988).

## Pharmacoinformatics approaches

Under pathological conditions, the low GABA concentration near a synapse induces a weaker activation of its receptors (provoking a delay in generating communication between pre- and post-synaptic neurons) thus, making the system more liable to the de-formation of new memories (Laviv et al., 2010). Thus, different biological assays including equilibrium binding assay, GABA uptake assay and GAT1 transport assay have been used to study the GABA transport through GAT1 in the presence of various antagonists in various cell lines including CHO, HEK, and *Xenopus* oocytes (Kragler et al., 2005, 2008; Pizzi et al., 2011).

Additionally, numerous attempts have also been made to identify the GATs inhibitors by using combined structure and ligand based strategies. Main focus was to remain on GAT1 as limited GAT1 inhibitory compounds failed to enter the clinical phase due to their impairment of motor activities and inability to cross the BBB (Falch et al., 1987). One of the successful inhibitor, Tiagabine, is the only FDA approved second generation GAT1 selective antiepileptic drug in the market with less toxicity however, certain side effects such as tremor, ataxia, asthenia and sedation are related to its pharmacological activity (Schwartzkroin, 2009). In general, Tiagabine is the derivative of nipecotic acid with the lipophilic chain attached to the protonated nitrogen of the piperidine ring of nipecotic acid at one end and di-thiophene rings substitutions at the other end (Genton et al., 2001). Various authors utilized pharmacoinformatics approaches to design selective inhibitors of GATs subtypes however, only handful of compounds could meet the selectivity and affinity criteria. Thus, less statistics are available about the potent inhibitors of GAT2, GAT3, and BGT1 as compared to GAT1 (Clausen et al., 2005a).

### Structure based studies

#### Homology modeling

Overall, a brief overview of different conformations of hGAT1 studied through X-ray crystallography technique in the bacterial and fly homolog has been presented in Table 1.

**Table 1:** Reported crystal structures of *Aa*LeuT and dDAT deposited in RCSB PDB.

**PDB code**	**Resolution (Å)**	**Conformation**	**Wild/ Mutated**	**Substrate**	**Inhibitor**	**Organism**	**Year**	**References**
2A65	1.65	Occluded-Out	Wild	L-Leu	–	A. *aeolicus*	2005	Yamashita et al., 2005
2Q6H	1.85	Occluded-Out	Wild	L-Leu	Clomipramine	A. *aeolicus*	2007	Singh et al., 2007
2Q72	1.70	Occluded-Out	Wild	L-Leu	Imipramine	A. *aeolicus*	2007	Singh et al., 2007
2QB4	1.90	Occluded-Out	Wild	L-Leu	Desipramine	A. *aeolicus*	2007	Singh et al., 2007
2QEI	1.85	Occluded-Out	Wild	L-Ala	Clomipramine	A. *aeolicus*	2007	Singh et al., 2007
2QJU	2.90	Occluded-Out	Wild	–	Desipramine	A. *aeolicus*	2007	Zhou et al., 2007
3F3A	2.00	Open-To-Out	Wild	–	L-Trp	A. *aeolicus*	2008	Singh et al., 2008
3F3C	2.10	Occluded-Out	Wild	*p*-F-L-Phe	–	A. *aeolicus*	2008	Singh et al., 2008
3F3D	2.30	Occluded-Out	Wild	L-Met	–	A. *aeolicus*	2008	Singh et al., 2008
3F3E	1.80	Occluded-Out	Wild	L-Leu	–	A. *aeolicus*	2008	Singh et al., 2008
3F48	1.90	Occluded-Out	Wild	L-Ala	*-*	A. *aeolicus*	2008	Singh et al., 2008
3F4I	1.95	Occluded-Out	Wild	L-Se-Met	*-*	A. *aeolicus*	2008	Singh et al., 2008
3F4J	2.15	Occluded-Out	Wild	Gly	*-*	A. *aeolicus*	2008	Singh et al., 2008
3GJC	2.80	Occluded-Out	Mutant	L-Leu	OG	A. *aeolicus*	2009	Quick et al., 2009
3GJD	2.00	Occluded-Out	Wild	L-Leu and OG	–	A. *aeolicus*	2009	Quick et al., 2009
3GWU	2.14	Occluded-Out	Wild	–	Sertraline	A. *aeolicus*	2009	Zhou et al., 2009
3GWV	2.35	Occluded-Out	Wild	–	R-fluoxetine	A. *aeolicus*	2009	Zhou et al., 2009
3GWW	2.46	Occluded-Out	Wild	–	S-fluoxetine	A. *aeolicus*	2009	Zhou et al., 2009
3MPN	2.25	Occluded-Out	Mutant	L-Leu	–	A. *aeolicus*	2010	Kroncke et al., 2010
3MPQ	2.25	Occluded-Out	Mutant	L-Leu	–	A. *aeolicus*	2010	Kroncke et al., 2010
3TT3	3.22	Inward-open &outward open	Wild	Substrate free (open-outward and apo inward)	–	A. *aeolicus*	2012	Krishnamurthy and Gouaux, 2012
4XP1	2.89	Open-To-Out	Wild	Dopamine	–	*D. melanogaster*	2015	Wang et al., 2015
4XP4	2.80	Open-To-Out	Wild	–	Cocaine	*D. melanogaster*	2015	Wang et al., 2015
4XP5	3.3	Open-To-Out	Wild	Cocaine analog-RTI55	*-*	*D. melanogaster*	2015	Wang et al., 2015
4XP6	3.1	Open-To-Out	Wild	–	methamphetamine	*D. melanogaster*	2015	Wang et al., 2015
4XP9	2.8	Open-To-Out	Wild	–	D-amphetamine	*D. melanogaster*	2015	Wang et al., 2015
4XPA	2.95	Partially occluded	Wild	3,4dichlorophenethylamine	–	*D. melanogaster*	2015	Wang et al., 2015
4XPB	3.05	Open-To-Out	Mutant	–	Cocaine	*D. melanogaster*	2015	Wang et al., 2015
4XPF	3.27	Open-To-Out	Mutant	–	RTI-55	*D. melanogaster*	2015	Wang et al., 2015
4XPG	3.21	Open-To-Out	Mutant	–	beta-CFT	*D. melanogaster*	2015	Wang et al., 2015
4XPH	2.9	Open-To-Out	Mutant	3,4dichlorophenethylamine	–	*D. melanogaster*	2015	Wang et al., 2015
4XPT	3.36	Open-To-Out	Mutant	3,4 dichlorophen ethylamine	–	*D. melanogaster*	2015	Wang et al., 2015

**Figure 4:**
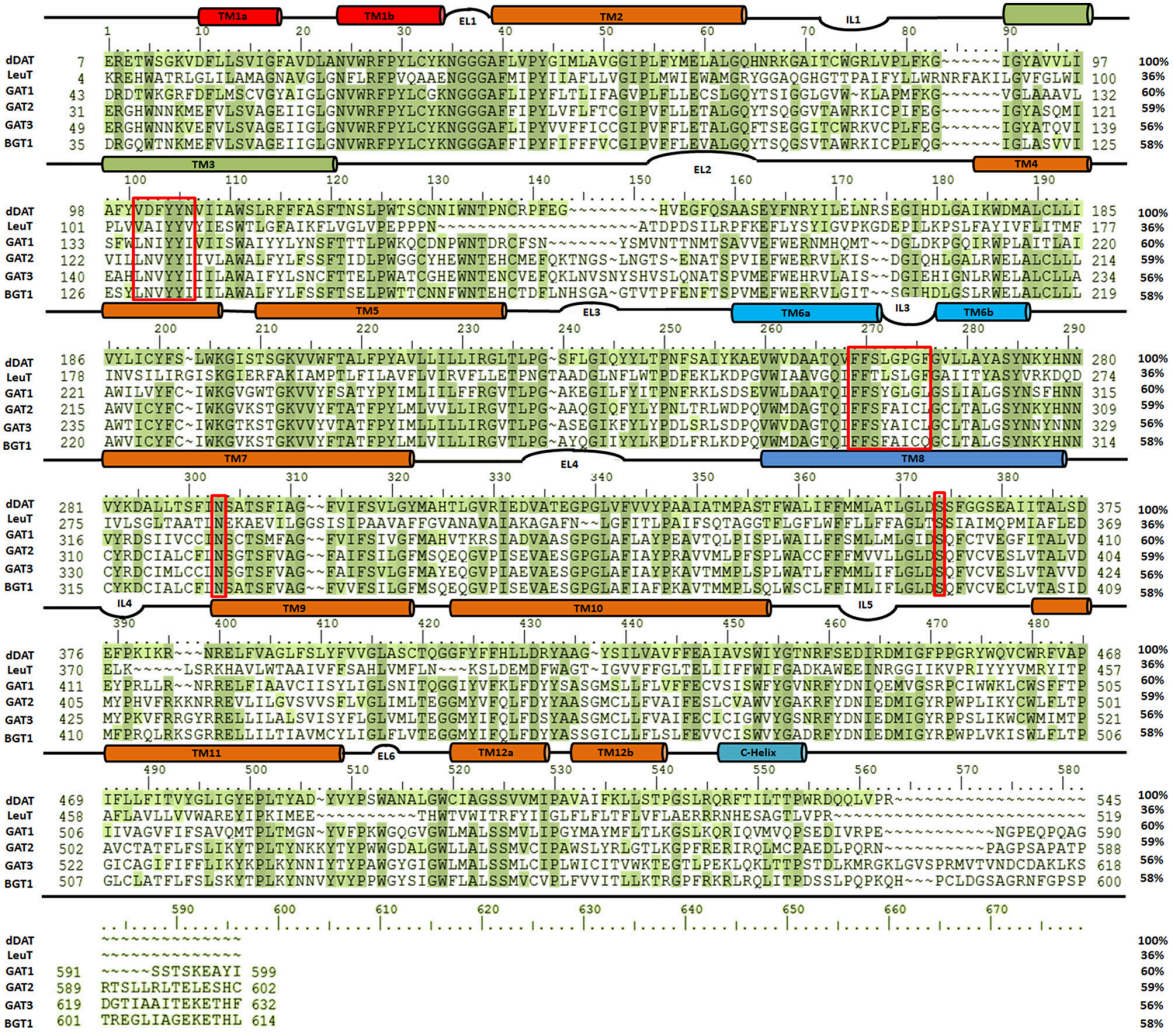
Sequence similarity among dDAT (UniProt ID: Q7K4Y6), *Aa*LeuT (UniProt ID: O67854) and all four subtypes of GATs [UniProt IDs: GAT1 (P30531), GAT2 (Q9NSD5), GAT3 (P48066), and BGT1 (P48065)]. The multiple sequence alignment (MSA) was performed using Schrodinger alignment tool (Schrödinger Release, 2017). The red boxes highlight the amino acid residues of the respective substrate binding sites in the aligned homologous sequences.

It has been elucidated that all four isoforms of GATs (GAT1-3 and BGT1) share >50% sequence similarity as shown in Figure 4. However, hGAT1 shares 60% sequence similarity with dDAT as compared to *Aa*LeuT (36%) which makes dDAT valuable template for structural modeling of hGATs and to resolve nature and shape of binding pocket, opening and closing conformations of GAT1 through further docking and molecular dynamic simulation (MD) studies.

In the last decade, homology models of different isoforms of GATs have been developed to understand their structural and functional characterization in humans. In this regard, Baglo and colleagues conducted homology modeling of the hGAT1 using *Aa*LeuT crystal structure as a template in three different conformations i.e., open-to-out (PDB ID: 3F3A), occluded-out (PDB ID: 2A65), and open-to-in (PDB ID: 3TT3). However, due to the difference in number of amino acid residues of EL2 among prokaryotes and eukaryotes maximum length of EL2 was not considered for model building (Baglo et al., 2013). The residues A61, I62, G63, L64, N66, S295, L300, S396, Q397, and C399 have been predicted to be involved in both GABA binding and transport. However, Dodd et al. (Dodd and Christie, 2007) and Anderson et al. (2010) have analyzed that the residues Y60, L136, G297, and T400 were specifically involved in GABA transport activity. The built homology models of GAT1 are discussed in detail in section Docking and Molecular Dynamics Simulations (MD) Studies with respect to amino acid residues involved in docking of ligands.

#### Docking and molecular dynamics simulations (MD) studies

Until now only two investigations have been carried out to computationally scrutinize the binding of substrate, two Na^+^ and a Cl^−^ ion in the S1 binding site of hGAT1 through molecular docking followed by molecular dynamics simulation studies. In addition to this, binding of such small molecules in GAT1 pocket allowed the researchers to predict the corresponding biological activities as well (Palló et al., 2007; Wein and Wanner, 2010).

Therefore, docking of small molecules into the binding pocket of hGAT1 provides a way to understand their mechanism along with the shape and nature of the binding core. Noticeably, in hGAT1 the coordination of one of the Na^+^ ion was observed with the carboxyl group of GABA. Moreover, GABA forms hydrogen bonds with the side chain hydroxyl group of Y140, to the main chain nitrogen atom of G65 and to the main chain oxygen of F294. The amine moiety of GABA in addition form ionic interactions with Y60 (Lovinger, 2010; Baglo et al., 2013).

**Figure 5:**
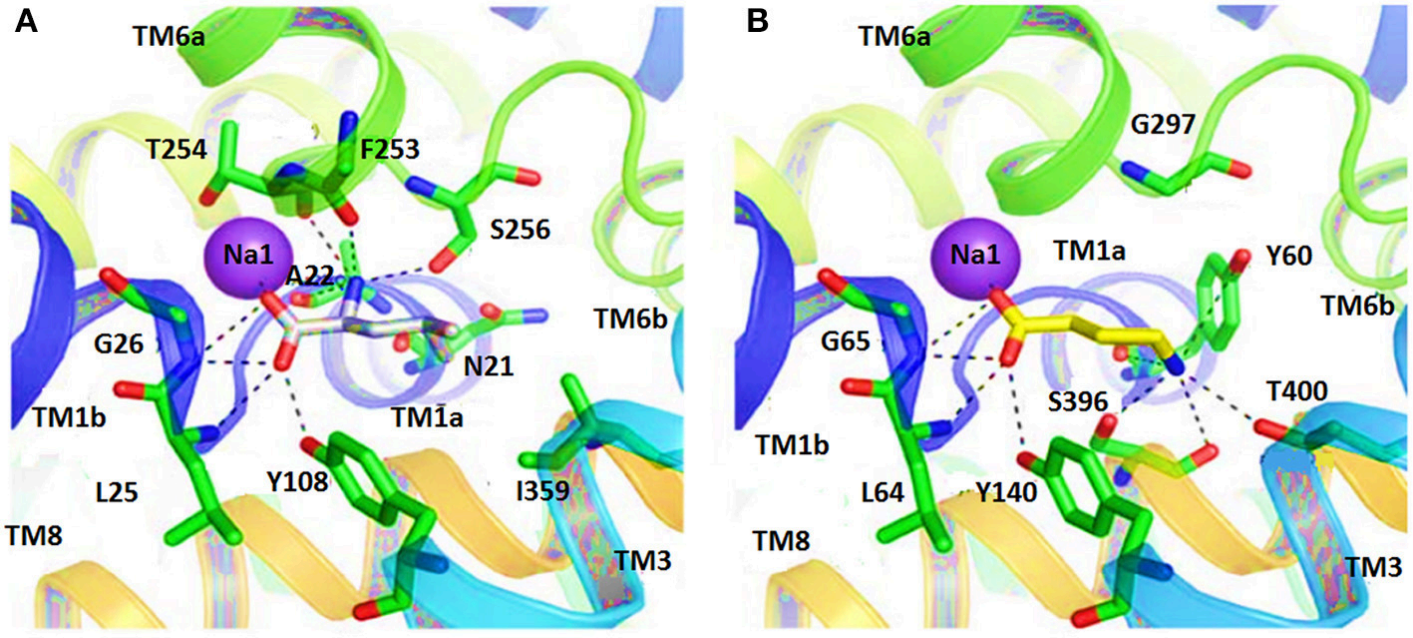
Comparison of ligand binding site residues in **(A)** Leucine bound LeuT and **(B)** GABA bound hGAT1 [Taken and modified from Wein and Wanner (2010)]. Residue numbers for the shown polar interactions in LeuT and hGAT1 is according to the respective transporters.

In another study, the binding pattern of substrates of hGAT1 and *Aa*LeuT i.e., GABA and leucine, respectively were analyzed. As both of the substrates possess carboxylic acid group, involved in interaction with the Na^+^ ion therefore, represented a very similar pattern of binding. In comparison to *Aa*LeuT, the carbon chain of the GABA adopted extended conformation in the binding pocket thus –NH of the GABA showed the hydrogen bond interaction with Y60 and G297 of hGAT1 as shown in Figure 5 (Wein and Wanner, 2010). Later on, small molecule inhibitors such as nipecotic acid, guvacine, 4-amino-isocrotonic, taurine, and 4-amino-2-hydroxybutanoic acid were also docked into the built hGAT1 model to probe their binding in hGAT1. The subsequent molecular dynamics (MD) calculations after flexible docking showed that the active site was not easily accessible either from the extracellular or cytoplasmic face because it was of very limited size hitherto, suggested that the large inhibitors bind in open-to-out conformation only (Wein and Wanner, 2010).

**Figure 6:**
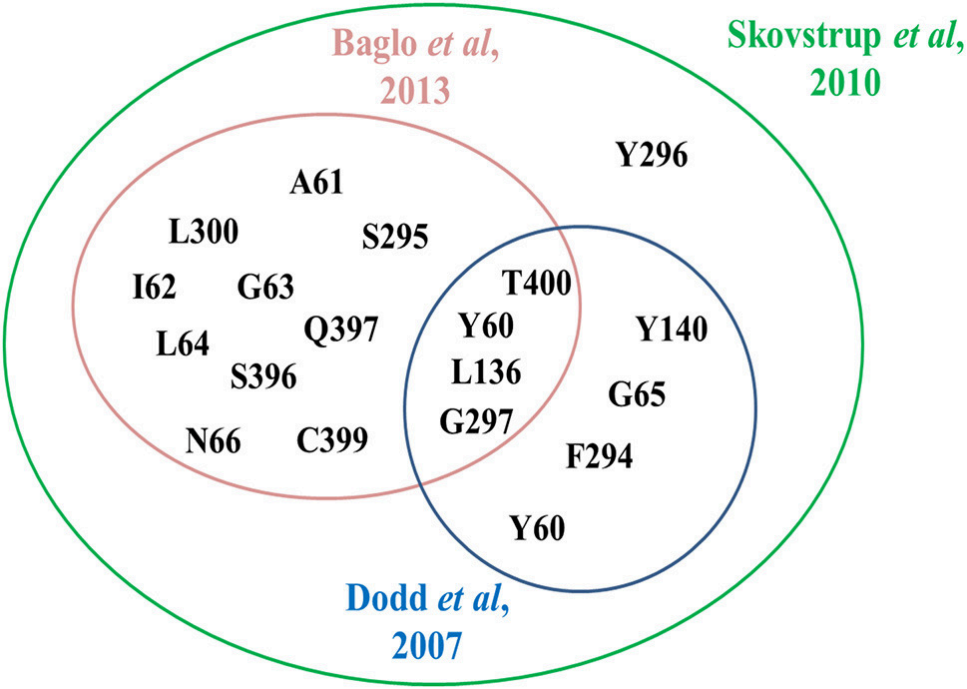
Comparison of specific and overlapping interacting amino acid residues at GABA binding site in GAT1 determined by Skovstrup et al. with the residues identified by Baglo et al. and Dodd et al.

In 2010, Skovstrup and colleagues studied the binding conformations of GABA, nipecotic acid and Tiagabine in occluded-out conformation of hGAT1. Figure 6 show a venn diagram of overlapping interacting amino acid residues in substrate binding site of GAT1 identified by previous researchers. It illustrates that T400, Y60, L136, and G297 amino acid residues play an important role in the binding of GABA and nipecotic acid derivatives (Yamashita et al., 2005; Gether et al., 2006; Dodd and Christie, 2007; Skovstrup et al., 2010; Baglo et al., 2013) however, Skovstrup et al. additionally reported the role of Y296 in the GABA binding.

**Figure 7:**
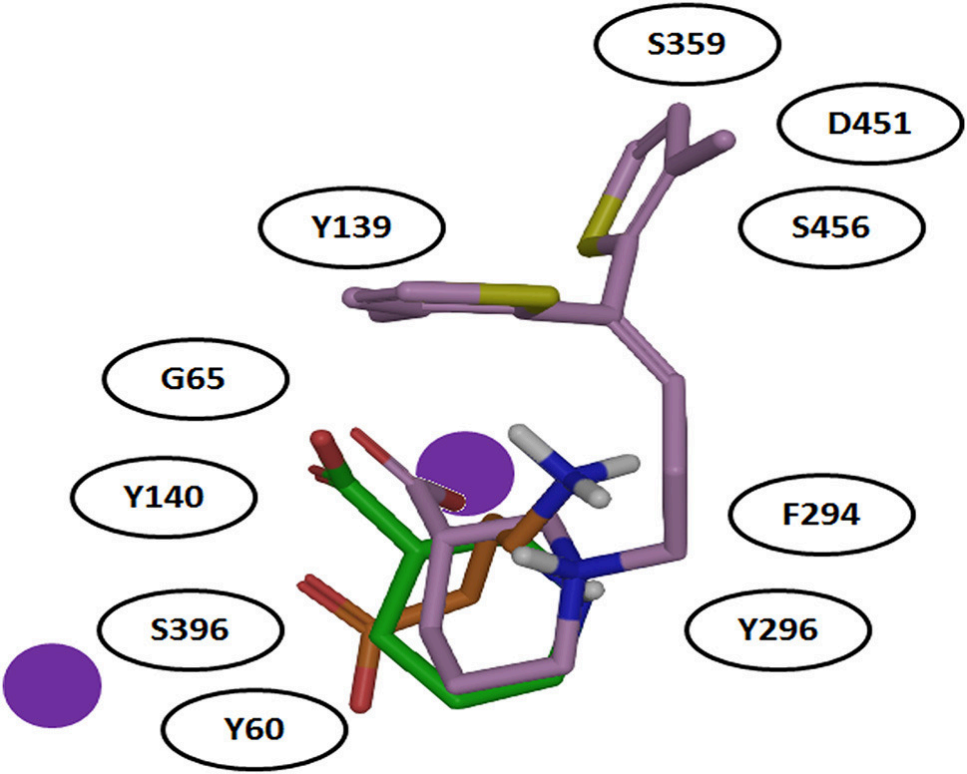
Comparison of binding mode of docked GABA, nipecotic acid and Tiagabine in GAT1 [taken and modified from Skovstrup et al. (2010)]. All of the three docked ligands have showed ionic interactions with one of the sodium ion present in the GAT1 binding pocket. The aromatic moieties of Tiagabine (purple) face the extracellular vestibule of GAT1 due to the presence of the linker lipophilic chain.

It was also hypothesized that the large aromatic moieties of GAT1 modulators are important for their inhibition activity. The attachment of large hydrophilic chains to the aromatic moieties may allow the inhibitor Tiagabine to face the extracellular vestibule of GAT1 in comparison to nipecotic acid (devoid of hydrophilic chain) which orients toward the cytoplasmic face of GAT1 (Figure 7) i.e., formation of hydrogen bond interaction between the protonated nitrogen of Tiagabine and F294(O) in occluded-out conformation. Moreover, all of the three compounds (i.e., GABA, nipecotic acid, and Tiagabine) showed electrostatic interaction with sodium ion while shared common polar interactions with Y60, Y140, and S396. However, the specific polar contacts (in case of GABA) were seen with Y296, G65 (in nipecotic acid), and F294 (in Tiagabine). On the other hand, MD simulations for open-to-out conformation of these compounds were also in agreement with the observations for occluded-out conformation (Skovstrup et al., 2010). This shows that occluded-out conformation requires major change in binding cavity for adjusting large inhibitors such as Tiagabine.

Later on, steered molecular dynamics (SMD) simulation approach was utilized to understand the whole mechanism of action of GABA in all the three GAT1 conformational states. Skovstrup and colleagues were successful in reorienting the occluded-out conformation into open-to-out and open-to-in conformations (Skovstrup et al., 2012). In case of reorientation to open-to-out conformation, the amino acid residues involved in the transfer of GABA from S1 site to temporary binding site (S2), located in the extracellular vestibule of GAT1, were determined. Before dissociation of GABA from S1 to S2 site the carboxylate group of GABA showed (i) intra-molecular interaction with amine of GABA (ii) ionic interaction with the Na1 and (iii) hydrogen bonding with Y60(O), G65(NH), Y140(OH), and S396(OH) (Skovstrup et al., 2010). However, after 8 ns of simulation, the amine of GABA and D451 from S2 site started water mediated interaction with each other. Moreover, R69 rearranged itself to form ionic interaction with GABA carboxylate through guanidinium to inhibit the drifting of GABA. The residues Y72 (located one helical turn above R69) and K76 (located one helical turn above Y72 and two helical turns above R69) took part in GABA binding after ~12 ns of MD simulation. The complex remained stable for around more 6 ns however, the GABA was fully solvated afterwards (at 19 ns time period, sticked in the extracellular vestibule) representing the open-to-out conformation of GAT1 (Skovstrup et al., 2010). While in case of open-to-in GAT1 conformation, the conformational change of TM6 results in the displacement of the residue Y60 which in turn disrupted the interaction between the carboxylate of GABA and Na1 of hGAT1. The residues R44, W47, F53, Q106, Y309, N310, and N314 were observed to be involved in the formation of intracellular gate. Additionally, E101 made ionic contact with amine group of GABA hitherto emancipated GABA into the cytoplasm. Therefore, the channels from S1 to S2 (dissociation and release of GABA in extracellular space) and S1 to cytoplasm have been recognized hydrophobic in nature. On the other hand, R-nipecotic acid showed similar dissociation effect as that of the GABA whereas; Tiagabine showed hydrophobic interactions with the residues of TM1 and TM6 in between two binding sites i.e., S1 and S2 (Skovstrup et al., 2012).

**Figure 8:**
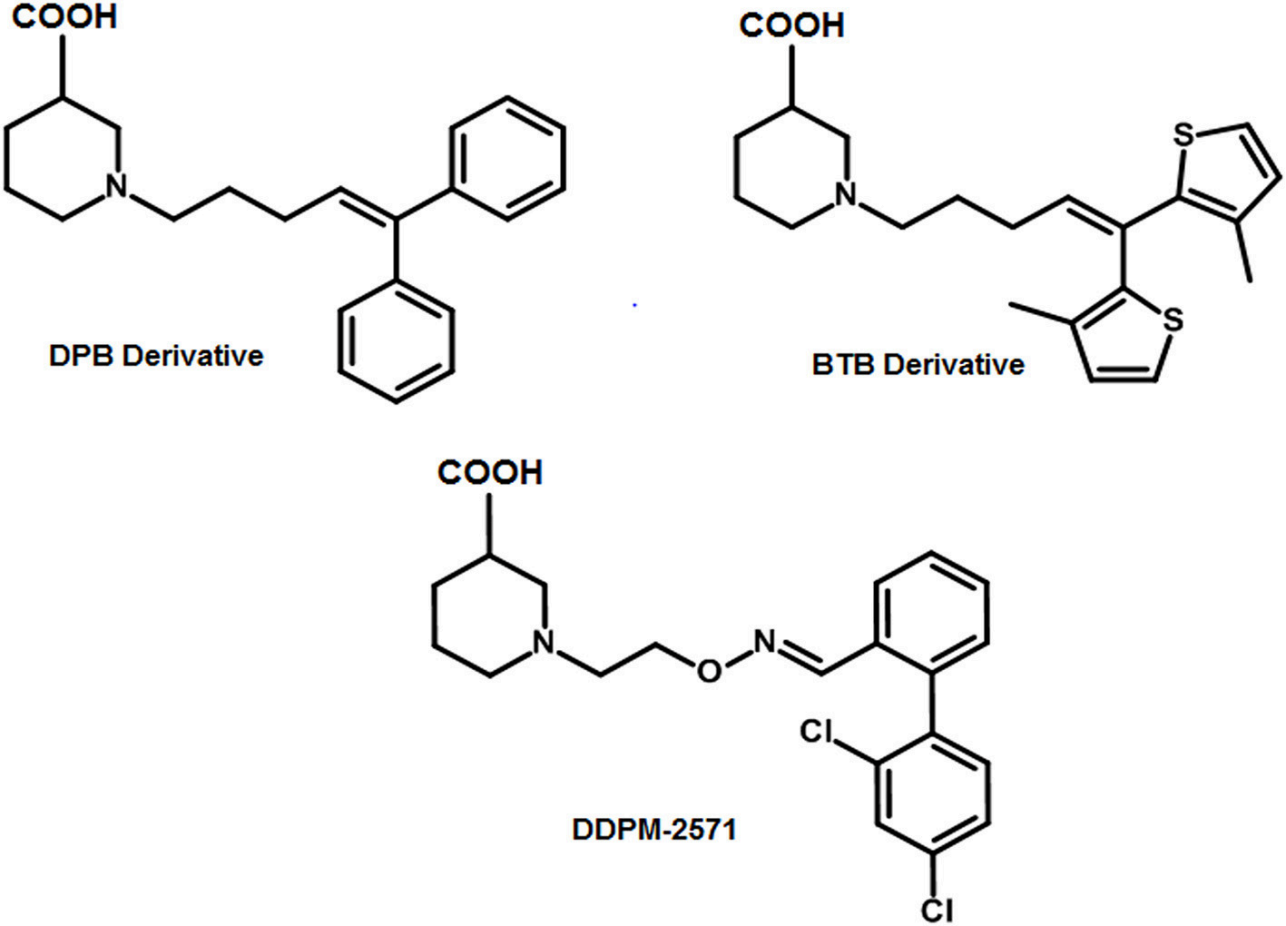
Examples of N-substituted nipecotic acid derivatives for the inhibition of GAT1 with DDPM-2571 (pIC_50_ = 8.29 ± 0.02) having pIC_50_ value comparable to the Tiagabine (7.43 ± 0.11).

R-nipecotic acid is known to be a medium-to-strong inhibitor of hGAT1 however; proline is known to be a weak inhibitor (Quandt et al., 2013). In 2016, Wein and colleagues synthesized a series of N-substituted 4,4-diphenylbut-3-en-1-yl (DPB) and 4,4-bis(3-methylthiophen-2-yl)but-3-en-1-yl (BTB) nipecotic acid and proline derivatives (examples shown in Figure 8). Interestingly in comparison to pure amino acids, the resultant BTB or DPB substituted amino acids showed similar binding affinities. On the other hand, docking of all these inhibitors in hGAT1 pocket has portrayed that the nitrogen atom of the pure amino acids is oriented toward the intracellular face of the hGAT1 whereas the nitrogen atom of the N-substituted BTB or DPB derivatives face the extracellular vestibule. This led to the finding that in order to augment the hGAT1 locking in open-to-out conformation the N-substituted amino acid derivatives are better option as compared to the pure amino acids (Wein et al., 2016).

Therefore, DDPM-2571 has been synthesized later, an N-substituted derivative of pyridine. DDPM2571 (pIC_50_ = 8.29 ± 0.02) showed comparative affinity to Tiagabine (pIC_50_ = 7.43 ± 0.11) when subjected to the hot plate test, formalin test and mouse models. In addition, DDPM2571 (shown in Figure 8) did not disrupt motor skills of the mouse models in lieu it has augmented the memory deficits. Thus, DDPM-2571 may be declared as a lead structure for the inhibition of seizures in hGAT1 as well (Sałat et al., 2017).

Recently, nipecotic acid derivatives with alkyne type spacer followed by the aromatic moiety have been synthesized. The comparison of Tiagabine and newly synthesized nipecotic acid derivatives showed a hydrogen bond interaction between protonated nitrogen and carbonyl carbon of F294 of hGAT1 (Lutz et al., 2017). Moreover, a binding mode hypothesis of nipecotic acid and N-diarylalkenyl piperidine analogs has been determined in newly developed hGAT1 model (template: dDAT, PDB ID: 4XP4) that may provide a structural basis to apprehend hGAT1 analogs binding and design. The identified binding site residues were in good agreement with already known roof and base residues of hGAT1 pocket (Sadia, 2018).

### Ligand based studies

From early 1980s, several attempts have been made to optimize lead structures of GATs inhibitors. Hereof, researchers attempted to employ amino acids, non-amino acids and their respective derivatives to develop GATs antagonists (Andersen et al., 2001). Among all, the bi-aromatic rings attached to the lipophilic moiety are of fundamental importance (Kragler et al., 2008) however, the underlying molecular mechanism of interaction of these lipophilic analogs with GABA uptake system is unknown (Stromgaard et al., 2009). A breakthrough in our understanding of GATs pharmacology came with the development of a nipecotic acid derivative with a di-aromatic substituent attached to the lipophilic chain. The resulting analog Tiagabine was found to be a potent, subtype specific and competitive inhibitor with a high affinity (IC_50_ = 0.049 μM) (Nakada et al., 2013). Later on, derivatives of these cyclic GABA analogs such as 4,4-diarylbutenyl, aminomethylphenols, tetrahydrobenzo-isoxazols, diaryloxime, pyrrolidine-2-acetic acid derivatives, and diarylvinyl ethers have been used to design and synthesize well-known specific inhibitors of GAT1 (Knutsen et al., 1999; Andersen et al., 2001; Zhao et al., 2005; Kragler et al., 2008; Pizzi et al., 2011).

**Figure 9:**
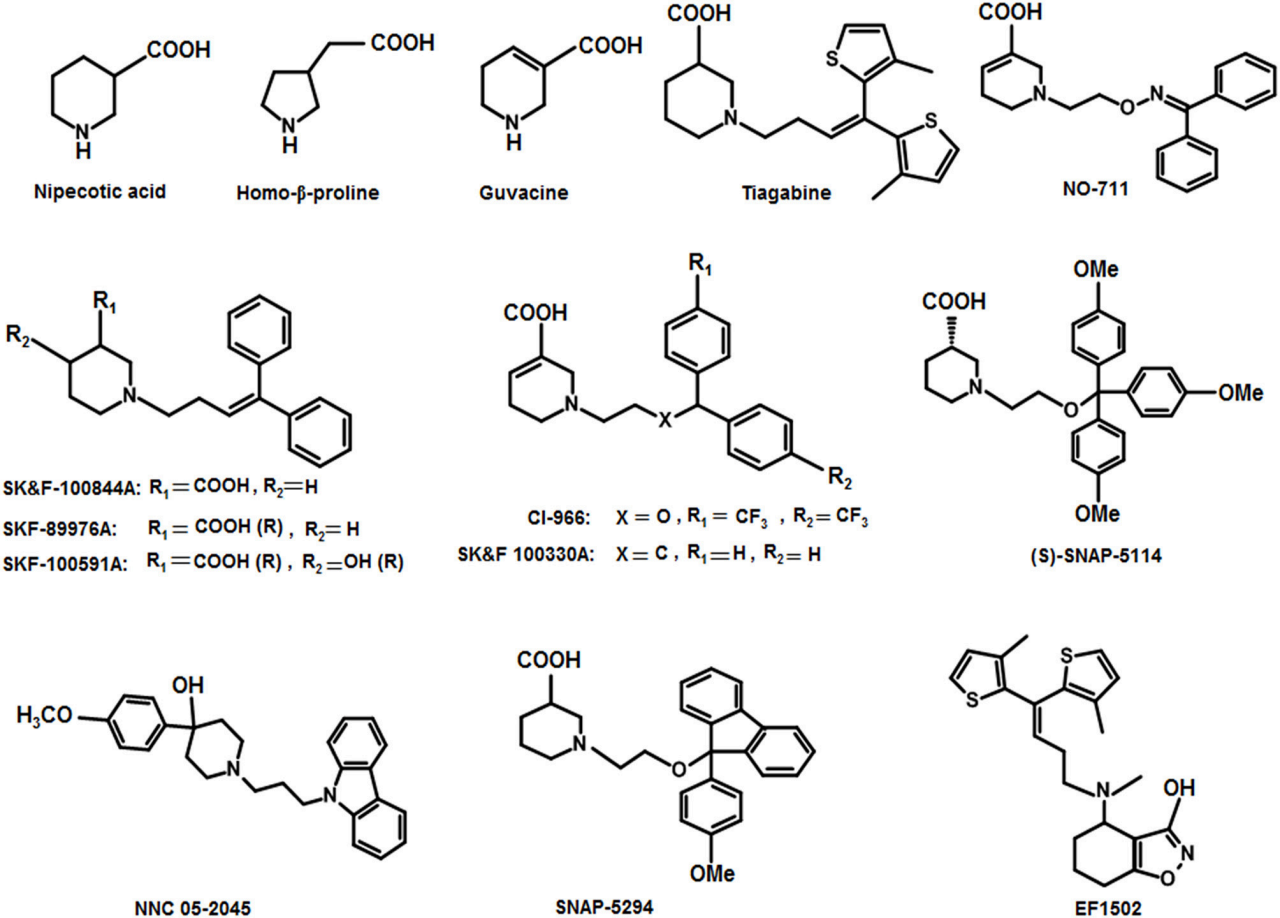
Chemical structures of well-known GAT inhibitors.

Thorough investigations of the compounds guvacine, proline and nipecotic acid led to the identification of the phenomenon that the addition of lipophilic side chains to these compounds results in the second generation compounds having ability to penetrate BBB. For example, SK&F 89976A (Murali Dhar et al., 1996), SKF-100591A (Zhao et al., 2005) SK&F 100330-A, CI-966 (Borden et al., 1994), NNC 711 (or NO 711), Tiagabine (highly selective for GAT1), SNAP-5294 (highly selective for GAT2) (Hack et al., 2011), (S)-SNAP-5114 (moderately selective for GAT3), NNC 05-2045, (poorly selective for BGT1), EF1502 (selective for GAT1/BGT1) etc (Figure 9). Normally, the lipophilic side chain is added onto the nitrogen atom of the parent molecule. The side chain addition has showed a significant increase in potency of many of the derivative inhibitors of GATs however, these compounds have not reached to the status of drugs (Pavia et al., 1992).

Andersen and colleagues synthesized novel tricyclic analogs from the **amino acids** nipecotic acid, guvacine, and homo-β-proline (Figure 9). The di-aryl groups were replaced with the tricyclic ring moieties and were further attached with the parent amino acid by the addition of variable length of hydrophilic chains, containing the electronegative moiety. However, this replacement decreased the potency of newly synthesized compounds with the exception of one derivative of homo-β-proline (HOM) that have showed 3-fold high potency (compound **1**), better ligand efficiency and hydrophilicity as compared to the parent compound. The Andersen group later extended the library of compound **1** like compounds by modifying the “A” and “R” substituents, resulting in moderate and poor inhibitors with a 0.18–40 μM affinity (Table 2). Later on, *in vivo* testing of compound **1** (IC_50_ = 0.05 μM) for neuronal [^3^H]-GABA uptake inhibition in mice also reveal its anticonvulsant activity (i.e., higher than the nipecotic acid and guvacine) (Andersen et al., 2001) approximately equivalent to the Tiagabine (IC_50_ = 0.049 μM) (Nakada et al., 2013).

**Table 2:** Derivatives of Compound 1 as modulators of hGAT1.

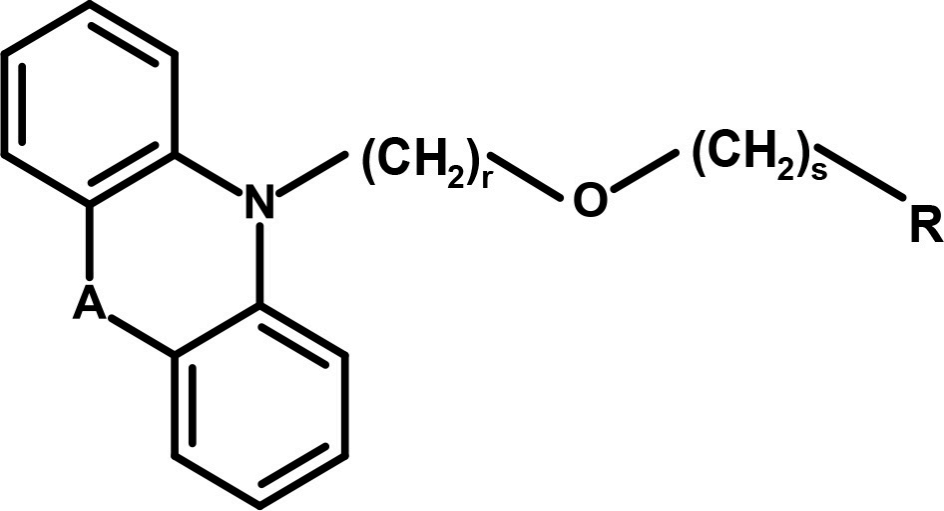					
**Compound 1**					
	**Bridging cell represented by “A”**	**r**	**s**	**R**	**GABA uptake IC**_50_ **(**μ**M)**
Compound 1	CH_2_CH_2_	2	2	homo-β-proline (HOM)	0.05
Derivatives of Compound 1	CH_2_CH_2_	2	2	(R)-nipecotic acid	0.18
	S	2	2	(R)-nipecotic acid	0.30
	O	2	2	(R)-nipecotic acid	14.6
	CH_2_	–	–	(R)-nipecotic acid	>40

Together, the following section provides a summary of the pharmacology of GATs inhibitors with emphasis on the recent advances in deciphering their role in hGAT1 binding pocket and corresponding biological activities.

#### Aminomethylphenols

**Figure 10:**
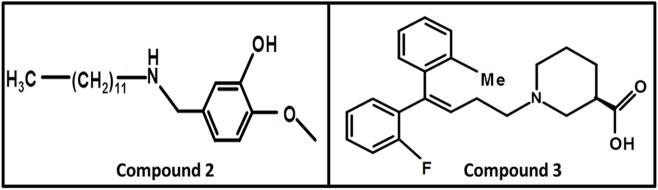
Chemical structures of GATs inhibitors having ability to cross BBB due to attachment of the linker lipophilic chain.

In 2008, Kragler and Wanner synthesized the **non-amino acid** aminomethylphenol derivatives and correlated their affinities against all GATs subtypes. The addition of the lipophilic side chain on the nitrogen of the aminomethylphenol molecule was applied to increase the flexibility of the compounds e.g., 5-n-dodecylaminomethyl-2-methoxyphenol (compound **2**, Figure 10). The compound **2** showed significant inhibition against both neuronal and glial [^3^H]-GABA uptake, although was subtype unspecific (IC_50_ values: GAT1 = 12.30 μM, GAT2 = 12.58 μM, GAT3 = 2.69 μM, BGT1 = 8.70 μM) (Kragler et al., 2008). Later on, Pizzi and colleagues investigated nipecotic acid analogs by incorporating methyl, chlorine, fluorine, and bromine on the ortho positions of the di-aromatic moieties attached to the lipophilic chain. Nevertheless, only the addition of methyl and flouro groups produced 4,4-diphenylbut-3-enyl derivative (compound **3**, pK_i_ = 7.83, Figure 10) using [3H]-Tiagabine radio ligand binding assay, with comparable affinity to Tiagabine (pK_i_ = 7.77) which also possess methyl group substituent at the ortho position of the thiophene rings (di-aromatic moieties) (Pizzi et al., 2011).

Nipecotic acid being a polar and hydrophilic compound is not a perfect GAT1 blocker (Stella et al., 2007) therefore, addition of N-(4,4-diphenyl-3-butenyl) hydrophilic moiety to the nipecotic acid resulted in the robust derivative SK&F89976A (Figure 9) with an about 20-fold improvement in affinity over nipecotic acid. However, replacement of N-(4,4-diphenyl-3-butenyl) lipophilic moiety with substituent 3,3-diphenylpropyl showed no *in vivo* activity at physiological pH, but improved *in vitro* activity (Stella et al., 2007; Wermuth, 2011).

In another study, 1F9 cells were observed to measure the proficiency of blockers SK&F89976A, SK&F100844A (4-methoxyphenyl derivative of SKF89976-A), and SK&F100330A (guvacine derivative) against GAT1 (Figure 9). Two of the three derivatives (SK&F100844A and SK&F89976A) possessed saturated piperidine rings however, SK&F100330A contained unsaturated piperidine ring with the biological activities of 10, 0.8, and 0.5 μM, respectively (Corey et al., 1994). Likewise, Yunger et al. also acknowledged the anticonvulsant activity of SK&F89976A, SK&F100330A, and SK&F100844A (IC_50_ = 0.20, 0.21, and 1.25 μM, respectively) in rats brain using [^3^H]-GABA uptake assay (Yunger et al., 1984). Later on, Braestrup synthesized a nipecotic acid derivative Tiagabine (NO 328) with the side chain addition of (R)-N-[4,4-Bis(3-methyl-2-thienyl)but-3-en-l-yl] to the nitrogen atom of the piperidine ring. Tiagabine was declared as a potential selective GABA inhibitor in astrocytes/neuronal cells and also a potential radio-ligand to check the concentration of GABA uptake (Braestrup et al., 1990). Later on, Tiagabine was renowned as a GAT1 selective inhibitor (Madsen et al., 2011).

Moreover, Yang synthesized a series of lipophilic di-aromatic derivatives of 3-ethoxy-4,5,6,7-tetrahydrobenzo[d]isoxazol-4-one by reductive amination of O-alkylatedracemic to obtain the astrocyte specific GABA uptake blockers; (R)-4-amino-4,5,6,7-tetrahydrobenzo[d]isoxazol-3-ol or (R)-exo-THPO (Table 3). In addition, *in vitro* analysis of their binding affinities against induced convulsions was carried out against all the GATs subtypes along with expression testing in three mediums/systems i.e., HEK cell lines, neurons, and astrocytes. Surprisingly, the obtained derivatives were more selective for the neuronal cells in comparison to the other two systems with the highest selective compound **5** ((RS)-4-[N-(1,1-diphenylbut-1-en-4-yl)amino]-4,5,6,7-tetrahydrobenzo[d]isoxazol-3-ol) having high binding affinity of 0.14 μM (Table 3). Other examples include compound **6** (IC_50_ = 34 μM, attached nitrogen in S-conformation) being a potent blocker of GAT2 whereas R-conformation of nitrogen atom in compound **6** (IC_50_ = 4 μM) showed subtype selectivity for GAT1 (Table 3) (Clausen et al., 2005b). Additionally, N -methyl-exo-THPO (4,5,6,7-tetrahydroisoxazolo [4,5-c]pyridin-3-ol) with binding affinity of 28 μM acted as astrocytic GABA transport blocker (Table 3, compound 7) (Yang and Rothstein, 2009).

**Table 3:** Chemical structures of exo-THPO derivatives along with inhibitory potency (IC_50_) values against hGAT 1.

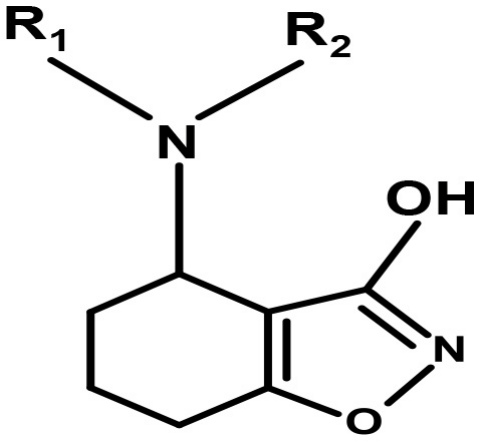			
**(R)-exo-THPO** *R_**1**_ and R_**2**_ = H (R-configuration)			
**Compound No**.	**R**_1_	**R**_2_	**IC**_50_ **(**μ**M)**
Compound 5	Ph_2_C = CH(CH_2_)_2_	–	0.14
Compound 6	C_14_H_15_S_2_	CH_3_	
N-atom in S-conformation			
N-atom in R-conformation			0.14
			4
Compound 7	CH_3_	–	28

#### Azetidine derivatives

**Figure 11:**
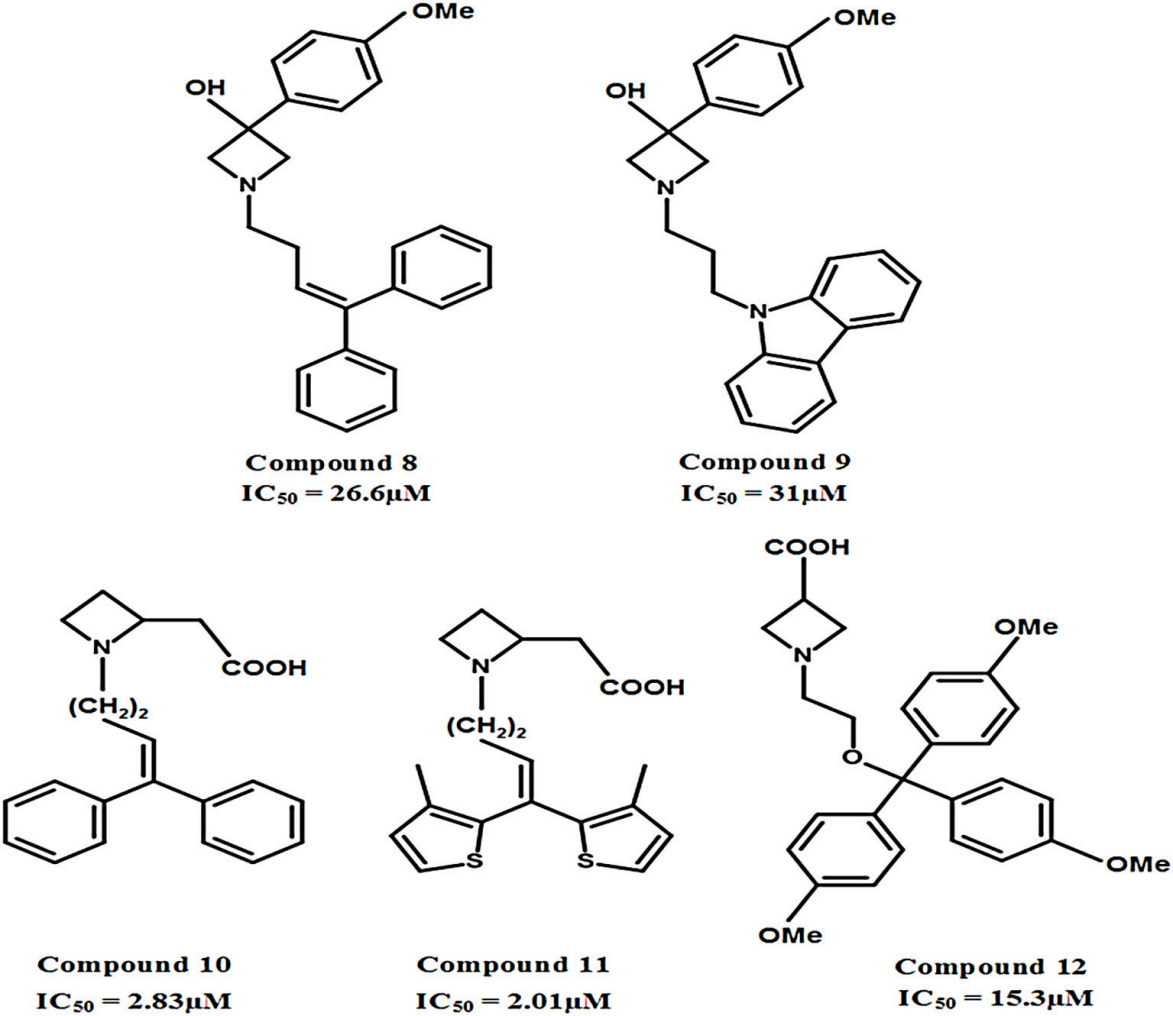
Chemical structures of azetidine derivatives as modulators of hGAT1. Compounds **10** and **11** were shown to be the most potent with respect to the other compounds in the series.

The carboxylic acid group attached at different positions (i.e., ortho, meta, or para) of the polar moiety of the GAT1 antagonists is known to play a crucial role toward high inhibitory potency (Zheng et al., 2004, 2006). However, another class of GATs inhibitors based on the bioisosteric substitution in place of carboxylic acid group with tetrazole ring was synthesized to evaluate the potential of the resultant azetidine derivatives. The subsequent derivatives displayed no effect on the GABA uptake which made tetrazole rings equipotent substitutors of carboxylic acid group. However, the substitution of piperidine ring in NNC-05-2045, one of the known GABA blocker, with the azetidine ring resulted in the potentially moderate azetidine derivatives of GAT1 e.g., 3-Hydroxy-3-(4-methoxyphenyl) (compound **8**, IC_50_ = 26.6 μM) and GAT3 (compound **9**, IC_50_ = 31 μM) as shown in Figure 11. Additionally, the insertion of 4,4-diphenylbutenyl or 4,4-bis(3-methyl-2-thienyl)butenyl moiety N-alkylated lipophilic side chains exhibited azetidine-2-ylacetic acid derivatives that ensured the highest activity against GAT1 (compound **10**, IC_50_ = 2.83 μM and compound **11**, IC_50_ = 2.01 μM). Whereas, the most active compound against GAT3 was the β-alanine analog 1-{2-[tris(4-methoxyphenyl)methoxy]ethyl}azetidine-3-carboxylic acid (compound **12**) with an IC_50_ = 15.3 μM (Figure 11) (Faust et al., 2010).

#### Aminomethyltetrazoles

**Figure 12:**
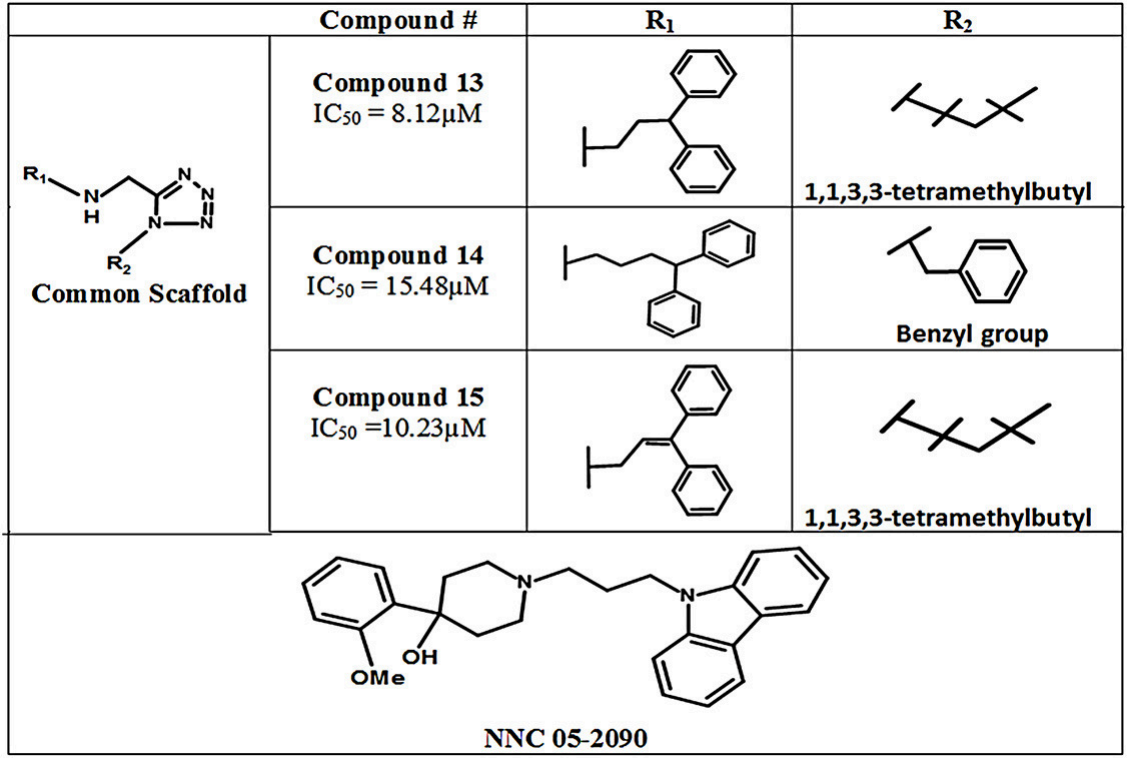
Chemical structures of aminomethyltetrazoles-type modulators of hGAT1.

In 2011, Glycine's mono- and di-substituted aminomethyltetrazole derivatives were evaluated for biological activity against all four subtypes of GATs in murine cells. 5-monosubsituted tetrazole blockers showed no contribution toward inhibition of the GABA whereas 1,5-disubstituted tetrazoles exhibited remarkable potential for the GAT2, GAT3, and GAT4 (BGT1 in humans) subtypes. For example, the highly selective di-substituted tetrazole derivative of GAT3 (compound **13**, IC_50_ = 8.12 μM) showed 4 and 12 folds higher selectivity in comparison to GAT4 and GAT1 subtypes, respectively (Figure 12). Until 2010, the GAT1 and GAT2 inhibitors were subtype unspecific due to the unavailability of detailed pharmacophore model of GAT2, which is still not completely solved. In this perspective, Schaffert's study provided a landmark in the identification of two new selective GAT2 inhibitors, having no impact on GAT1 activity i.e., compound **14** and compound **15** (IC_50_ = 15.48 and 10.23 μM, respectively). Moreover, the biological activity of Compound **15** was approximately similar to the activity of NNC-05-2090 i.e., IC_50_ = 8.12 μM (Figure 12) (Schaffert et al., 2011).

#### QSAR studies

So far, limited three dimensional-quantitative structure activity relationship (3D-QSAR) studies based on comparative molecular field analysis (CoMFA) and 2D-QSAR study on GAT1 have been conducted. Zheng et al., in 2004 and later in 2006 developed 3D-QSAR models for N-diarylalkenyl-piperidinecarboxylic acid analogs. It was hypothesized that either one or two of the aryl rings substituted with bulky phenoxymethyl and benzyloxymethyl group in the ortho position might improve the GAT1 inhibitory activity. Moreover, negative groups e.g., carboxylic acid meta position with respect to nitrogen atom of the piperidine ring displayed greater potency for the interaction of inhibitors with GAT1 and both steric and electronic factors were also shown to be important (Zheng et al., 2004, 2006).

Later on, Jurik et al. performed 2D-QSAR study on 162 nipecotic acid and guvacine derivatives with pIC_50_ = >7.0. Four different sets of descriptors including weinerPol, opr_brigid, 16 physicochemical descriptors, 32 van der Waals surface area (VSA) descriptors were used to build the model. In this respect, contingency matrix and VSA descriptors turned out to be well-suited to describe the dataset. Moreover, as 2D-QSAR is a versatile method for capturing SAR information, therefore the test compounds were easily differentiated as active ones having ortho-substitution in the linker region of the derivatives of nipecotic acid from the inactive compounds (Jurik et al., 2013).

**Figure 13:**
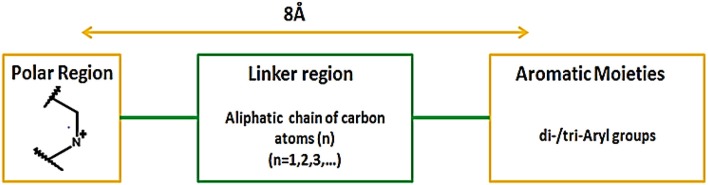
Favorable distance between the aromatic moieties and protonated nitrogen atom of the polar region within the common scaffold of hGAT1 antagonist to achieve maximum inhibition of hGAT1, adopted from Hirayama et al. (2001).

In addition, Hirayama and colleagues utilized pharmacophoric approach for the development of small molecule hSGLT1 and GAT1 inhibitors. Nipecotic acid derivatives, baclofen, saclofen, nortriptyline and SKF89976A compounds were used for the development of GAT1 pharmacophore model. The best pharmacophore model consisted of 1 hydrogen bond unfavorable region, 3 hydrogen bond donors and acceptors and 1 hydrogen bond donor site that plays a critical role in interaction between GAT1 and inhibitors. Moreover, it has been demonstrated that large aromatic or hydrophobic moieties of GAT1 inhibitors are separated at a distance of 8Å from the protonated nitrogen atom in the polar moiety (Figure 13). Overall, the GAT1 inhibitor's aromatic moieties binding position resides coplanar ~8Å from the substrate (GABA) binding site and is responsible for the inhibition of translocation process (Hirayama et al., 2001).

Recently, a GRIND model of GAT1 antagonists was developed using flexible alignment by pharmacophore mapping approach. The model represent good statistics at second cycle of Fractional Factorial Design algorithm (FFD2) (Palló et al., 2007) with correlation coefficient (*r*^2^) of 0.75. According to the model, two hydrogen bond acceptors (N1), one hydrogen bond donor (O) and one hydrophobic region (DRY) at certain distances from each other play an important role in achieving high inhibitory potency against hGAT1 (Sadia, 2018).

Briefly, the past decade has witnessed a paradigm shift in drug discovery with the help of computer aided drug design approaches. In this regard, the combine use of ligand based and structure based studies for the identification of GAT1 antagonists has bridged the gap between the ligands and transporter interactions. From the current review on GAT1, it has been deciphered that the hydrophobic region of GAT1 pocket allows the adjustment of the aromatic moieties of the GAT1 antagonists and sodium ion (Na1) of GAT1 is involve in making electrostatic interaction with the acidic group (most commonly COOH group) attached to the polar moiety. In addition, protonated nitrogen atom of polar region of GAT1 antagonists also plays an important role in interaction with F294/S295 of GAT1. In summary, over the short course of recent advances made for determining the mechanistic models of hGAT1, it might be expected that this progress will accelerate in the upcoming years and will serve as a fuel for the detailed insights of membrane transporter proteins. This should not only include the availability of high resolution X-ray structure of hGAT1 but also the development of new experimental protocols followed by the structure determination of other members of SLC6 family with more optimized computational models and methods.

## Outlook summary

Knowledge of the structure and function of GABA transporters continues to increase due to recent advancements in structural biology. In molecular mechanism perspective, the efforts to understand the structure and function of GATs are mainly compromised due to lack of crystal structure of mammalian GATs. However, the crystal structures of bacterial and fly homologs of GATs aids to comprehend the pharmacology of GATs. Until now, only a single GAT1 selective FDA approved drug Tiagabine is available against one of the most notable neurological disorder epilepsy that is caused due to dysregulation of GAT1. Various molecular modeling studies reported that one of the sodium ions in binding pocket of GAT1 form electrostatic interactions with Tiagabine. This may depict the importance of one sodium ion in the translocation cycle of hGAT1. Moreover, the residues G65 and Y140 of GAT1 are also observed crucial for the formation of hydrogen bond either with the docked substrate or inhibitors. Overall, the binding hypothesis of Tiagabine and its derivatives suggests that carboxylic acid moiety in the basic scaffold may contribute positively in achieving high inhibitory potency (IC_50_) against hGAT1. However, substitution of large functional groups on the thiophene rings (aromatic moieties) of Tiagabine may result in less potent GAT1 inhibitors. Therefore, this could provide a rationale to design more potent GAT1 inhibitors to mediate fast inhibitory neurotransmission.

## Author contributions

SZ and IJ conceived and designed the paper, figures and/or tables, reviewed drafts of the paper.

### Conflict of interest statement

The authors declare that the research was conducted in the absence of any commercial or financial relationships that could be construed as a potential conflict of interest.
